# MicroRNA-21 induces resistance to the anti-tumour effect of interferon-*α*/5-fluorouracil in hepatocellular carcinoma cells

**DOI:** 10.1038/sj.bjc.6605958

**Published:** 2010-10-26

**Authors:** Y Tomimaru, H Eguchi, H Nagano, H Wada, A Tomokuni, S Kobayashi, S Marubashi, Y Takeda, M Tanemura, K Umeshita, Y Doki, M Mori

**Affiliations:** 1 Department of Surgery, Graduate School of Medicine, Osaka University, Suita, 2-2 Yamadaoka E-2, Osaka 565-0871, Japan; 2 Division of Health Sciences, Graduate School of Medicine, Osaka University, Suita, Osaka, Japan

**Keywords:** hepatocellular carcinoma (HCC), interferon-*α* (IFN-*α*), 5-fluorouracil (5-FU), miR-21, phosphatase and tensin homologue (PTEN), programmed cell death 4 (PDCD4)

## Abstract

**Background:**

We reported recently the clinical efficiency of interferon (IFN)-*α*/5-fluorouracil (5-FU) combination therapy in advanced hepatocellular carcinoma (HCC). However, prediction of the response to the combination therapy remains unsatisfactory. The aim of this study was to investigate the anti-tumour effects of microRNA (miR)-21 on the sensitivity of HCC cells to IFN-*α*/5-FU and whether miR-21 can be used as a predictor of the response to such therapy in HCC.

**Methods:**

Changes in the sensitivity of HCC cells (PLC/PRF/5 and HepG2) to IFN-*α*/5-FU were examined after transfection with pre-miR-21 or anti-miR-21. The correlation between miR-21 expression level, evaluated by qRT–PCR, and response to the therapy was also investigated in clinical HCC specimens.

**Results:**

Hepatocellular carcinoma cells transfected with pre-miR-21 were significantly resistant to IFN-*α*/5-FU. Annexin V assay showed that the percentage of apoptotic cells was significantly lower in cells transfected with pre-miR-21 than control cells. Transfection of anti-miR-21 rendered HCC cells sensitive to IFN-*α*/5-FU, and such sensitivity was weakened by transfection of siRNAs of target molecules, PETN and PDCD4. miR-21 expression in clinical HCC specimens was significantly associated with the clinical response to the IFN-*α*/5-FU combination therapy and survival rate.

**Conclusions:**

The miR-21 in HCC cell lines and clinical HCC samples is a significant modulator of the anti-tumour effect of IFN-*α* and 5-FU. This suggests that miR-21 is a potentially suitable marker for the prediction of the clinical response to the IFN-*α*/5-FU combination therapy.

Hepatocellular carcinoma (HCC) is one of the most common malignancies worldwide. The prognosis of patients with advanced HCC remains poor, particularly in patients with tumour thrombi in the major trunk of the portal vein (Tanaka *et al*, 1996; Asahara *et al*, 1999). In such patients, conventional therapies have no clinical impact because of poor efficacy and possible complications (Furuse *et al*, 1997; Lee *et al*, 1997). Accordingly, new therapeutic approaches are needed for patients with advanced HCC.

Several studies have reported encouraging results for the therapeutic effects of the interferon (IFN)-based combination chemotherapy in HCC, compared with unsatisfactory results of IFN-*α* monotherapy (Urabe *et al*, 1998; Chung *et al*, 2000; Patt *et al*, 2003; Obi *et al*, 2006; Uka *et al*, 2007; Ueshima *et al*, 2008). We have also reported the clinical efficiency of IFN-*α* and 5-fluorouracil (5-FU) (IFN-*α*/5-FU) combination therapy for advanced HCC and the mechanism of its anti-tumour effect (Eguchi *et al*, 2000; Sakon *et al*, 2002; Yamamoto *et al*, 2004; Kondo *et al*, 2005; Ota *et al*, 2005; Nakamura *et al*, 2007; Wada *et al*, 2007, 2009; Damdinsuren *et al*, 2007a, 2007b; Nagano *et al*, 2007a, 2007b; Noda *et al*, 2009). These studies showed that IFN-*α* suppresses the proliferation of all type I IFN receptor type 2 (IFNAR2)-positive cancer cell lines *in vitro*, and that the expression of IFNAR2 in HCC tissues was significantly associated with clinical response to the IFN-*α*/5-FU combination therapy. These results indicate that IFNAR2 expression might be useful in the prediction of the clinical response to the combination therapy (Ota *et al*, 2005; Nagano *et al*, 2007a). However, the same studies also included several patients who were positive for IFNAR2 expression but did not show good clinical response, suggesting that the clinical response to the therapy cannot be predicted satisfactorily only by the expression of IFNAR2 (Ota *et al*, 2005; Nagano *et al*, 2007a). Accordingly, it is necessary to find novel biological markers that can more accurately predict the clinical response to the IFN-*α*/5-FU therapy.

MicroRNA (miR) is a small noncoding RNA gene product known to modulate the gene expression post-transcriptionally by negatively regulating the stability or translational efficiency of its target mRNAs (Bartel, 2004; Calin and Croce, 2006a). miRs control a wide array of biological processes, including cell differentiation, proliferation, and apoptosis. Aberrant expression of miRs has been widely reported in human cancers with both up- and downregulation detected in neoplastic cells compared with their normal counterparts (Croce and Calin, 2005; Calin and Croce, 2006b). Recently, some investigators reported a correlation between miRs expression and chemoresistance in several types of cancers. For example, Fujita *etal* (2008) reported that the expression of miR-34a attenuated chemoresistance to an anticancer drug in prostate cancer cells. Furthermore, the expression of miR-122 was also reported to be significantly associated with the sensitivity to sorafenib and doxorubicin (Bai *et al*, 2009; Fornari *et al*, 2009). Among these previous reports of correlation of miRs expression to chemoresistance, miR-21, which is reported to be increased in many cancers including HCC, is one of the most common miRs related to chemoresistance (Volinia *et al*, 2006; Meng *et al*, 2007). For example, it was reported that the miR-21 reduced the sensitivity to gemcitabine in cholangiocarcinoma cells (Meng *et al*, 2006). Also in glioblastoma cells, the miR-21 is reported to contribute to VM-26 resistance (Li *et al*, 2009). Furthermore, several studies reported a significant association between miR-21 expression and chemoresistance to gemcitabine in pancreatic cancer cells (Moriyama *et al*, 2009; Park *et al*, 2009).

In this study, we first examined the effects of miR-21 expression level in HCC cell lines on their sensitivity to IFN-*α* and 5-FU, and confirmed that miR-21 induced resistance to these chemotherapeutic agents. In the second part of the study, the expression level of miR-21 in human HCC tissue samples was significantly associated with the clinical response to the IFN-*α*/5-FU combination therapy.

## Materials and methods

### HCC cell line

The human HCC cell lines, PLC/PRF/5, HuH7, HLE, HLF, and HepG2, were obtained from the Japan Cancer Research Resources Bank (Tokyo, Japan). They were maintained in Dulbecco's modified Eagle's medium supplemented with 10% fetal bovine serum, 100 U per ml penicillin and 100 mg per ml streptomycin at 37 °C in a humidified incubator with 5% CO_2_ in air.

### Drugs and reagents

Purified human IFN-*α* and 5-FU were kindly supplied by Otsuka Pharmaceutical Co. (Tokyo, Japan) and Kyowa Hakko Kirin Co. (Tokyo, Japan), respectively. Monoclonal mouse anti-human phosphatase and tensin homologue (PTEN) antibody (Santa Cruz Biotechnology, Inc., Santa Cruz, CA, USA) and polyclonal rabbit anti-human programmed cell death 4 (PDCD4) antibody (Abcam Inc., Cambridge, MA, USA) were used for western blot analysis and immunohistochemistry.

### Transfection

microRNA-21 precursor (pre-miR-21), antisense miR-21 inhibitor (anti-miR-21), *PTEN* siRNA, *PDCD4* siRNA, and their negative control oligonucleotides were obtained from Ambion Inc. (Austin, TX, USA). These were used to transfect HCC cells by using siPORT NeoFx (Ambion Inc.) according to the instructions provided by the manufacturer. The transected cells were resuspended and cultured in regular culture medium for 48–72 h before analysis.

### Patients and specimens

The study subjects were 30 patients with advanced HCC and recruited as described previously (Nagano *et al*, 2007a). All patients had multiple liver tumours in both lobes and tumour thrombi in the main trunk of the portal vein, and each underwent palliative reduction surgery with tumour thrombectomy of the main trunk of the portal vein at the Osaka University Hospital between 1999 and 2004. The IFN-*α*/5-FU therapy for the remnant multiple liver tumour was applied postoperatively, as described previously (Ota *et al*, 2005; Nagano *et al*, 2007a). Patients were followed after surgery with postoperative follow-up period of 18.2±19.7 months (mean±s.d.). The clinical response to the therapy was evaluated according to the criteria of the Eastern Cooperative Oncology Group (Oken *et al*, 1982). On the basis of the clinical response, responders were defined as patients with complete response or partial response, and non-responders were defined as patients with stable disease or progressive disease. The study protocol was approved by the Human Ethics Review Committee of Osaka University Hospital and a signed consent form was obtained from each patient.

### RNA extraction

Total RNA and miR fractions were isolated from tissue samples and cell lines by TRIzol agent (Invitrogen, Carlsbad, CA, USA), and the quality of the RNA was assessed with a NanoDrop ND-1000 spectrophotometer (NanoDrop Technologies, Wilmington, DE, USA) at 260 and 280 nm (A260/280).

### Real-time quantitative reverse transcription-PCR for miR expression

Reverse transcription (RT) reaction and real-time quantitative RT-PCR (qRT–PCR) were performed using Taqman human miR assay kit (Applied Biosystems, Foster City, CA, USA) according to the instructions supplied by the manufacturer. The expression of the target miR was normalised relative to that of the internal control, RNU48. Data were analysed according to the comparative Ct method (Schmittgen *et al*, 2004).

### Real-time qRT-PCR for mRNA expression

Reverse transcription reaction was performed with SuperScript II (Invitrogen) on the basis of the protocol provided by the manufacturer, and qRT–PCR was performed as described previously (Kondo *et al*, 2005). The expression of the target gene was normalised relative to the expression of *porphobilinogen deaminase (PBGD)*, which was used as an internal control. The designed PCR primers were as follows: *matrix metalloproteinase (MMP)-2* forward primer, 5′-TGGCGATGGATACCCCTTT-3′ *MMP-2* reverse primer, 5′-TTCTCCCAAGGTCCATAGCTCAT-3′ *MMP-9* forward primer, 5′-CCTGGGCAGATTCCAAACCT-3′ *MMP-9* reverse primer, 5′-GCAAGTCTTCCGAGTAGTTTTGGAT-3′ *MMP-11* forward primer, 5′-TGACTTCTTTGGCTGTGCC-3′ *MMP-11* reverse primer, 5′-GTTGTCATGGTGGTTGTACCC-3′ *PBGD* forward primer, 5′-TGTCTGGTAACGGCAATGCGGCTGCAAC-3′ *PBGD* reverse primer, 5′-TCAATGTTGCCACCACACTGTCCGTCT-3′.

### Western blot analysis

Cells grown to semiconfluence were lysed in RIPA buffer (25 mM Tris (pH 7.5), 50 mM NaCl, 0.5% sodium deoxycholate, 2% Nonidet P-40, 0.2% sodium dodecyl sulphate, 1 mM phenylmethylsulphonyl fluoride and 500 KIE per ml Trasylol, proteinase inhibitor (Bayer, LeverKusen, Germany)). Western blot analysis was carried out as described previously (Kondo *et al*, 2005).

### Growth-inhibitory assay

Inhibition of cell growth in the presence of chemotherapeutic agents was assessed by the 3-(4-,5-dimethylthiazol-2-yl)-2,5-diphenyl tetrazolium bromide (MTT) (Sigma-Aldrich Co., St Louis, MO, USA) assay as described previously (Eguchi *et al*, 2000). Briefly, the cells were incubated for 72 h under various concentrations of IFN-*α* and 5-FU. After re-incubation for 4 h with MTT solution, acid-isopropanol was added to dissolve the resultant formazan crystals. The absorbance of the plate was measured in a microplate reader at a wavelength of 570 nm with a 650 nm reference, and the results were expressed as the percentage of absorbance relative to untreated controls.

### Annexin V assay

The binding of Annexin V was used as a sensitive method for measuring apoptosis, as described previously (Nakamura *et al*, 2007). At 24 h after treatment, cells were stained by Annexin V-FITC and propidium iodide (PI) (BioVision Research Products, Mountain View, CA, USA), and analysed on a FACS Calibur (BD Biosciences, Franklin Lakes, NJ, USA). Annexin V-positive and PI-negative cells considered as early apoptotic cells were used for the assessment of apoptosis in the study (Lugli *et al*, 2005).

### Immunohistochemistry

Immunohistochemical staining for PTEN and PDCD4 in the above-mentioned 30 HCC samples was performed by the method described previously (Kondo *et al*, 2005). Briefly, after deparaffinisation and blocking, the sections were incubated overnight at 4 °C with the antibody. The sections were counterstained with Meyer's haematoxylin. The PTEN and PDCD4 expression, defined as the presence of specific staining in the cytoplasm of cancer cells, was evaluated as positive or negative.

### Statistical analysis

Data were expressed as mean±s.d. Clinicopathological parameters were compared using the *χ*^2^-test, and continuous variables were compared using the Student's *t*-test. Survival curves were computed using the Kaplan–Meier method, and differences between survival curves were compared using the log-rank test. A *P*-value less than 0.05 denoted the presence of a statistically significant difference. Statistical analysis was performed using StatView (version 5.0, SAS Institute Inc., Cary, NC, USA).

## Results

### microRNA-21 expression is upregulated in tumoural tissue compared with non-tumoural tissue in HCC patients

**Figure 1:**
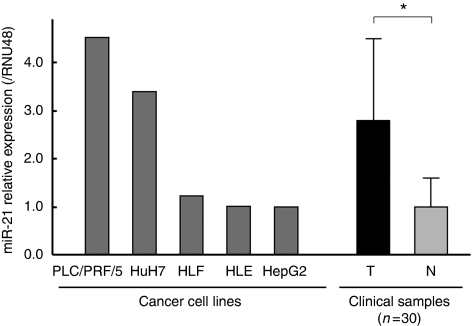
The expression level of miR-21 in five HCC cell lines including PLC/PRF/5, HuH7, HLE, HLF, and HepG2, and clinical samples from 30 patients with advanced HCC. The miR-21 expression was normalised by the average expression in non-tumoural tissues. The expression in tumoural tissue was significantly higher than in non-tumoural tissue (^*^P<0.05). Data are mean±s.d. T =tumoural tissue; N= non-tumoural tissue.

The expression of miR-21 was examined in tumoural tissue and non-tumoural tissue of the 30 patients with advanced HCC and also in the HCC cell lines. The expression in tumoural tissue was significantly higher compared with non-tumoural tissue, as reported previously by Meng *et al* (2007) (*P*<0.0001) (Figure 1). The expression in the HCC cell lines varied as shown in Figure 1.

### Transfection of pre-miR-21 induces resistance to IFN-*α* and 5-FU

**Figure 2:**
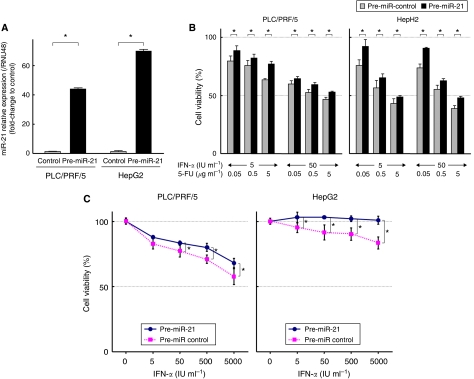

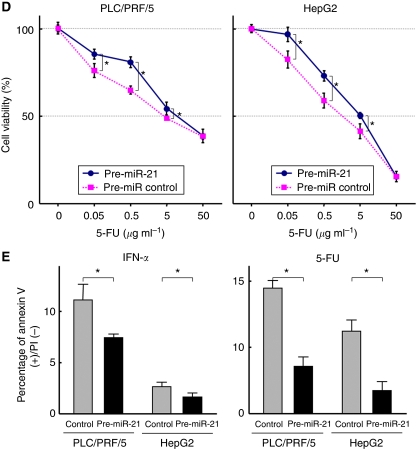
Transfection of pre-miR-21 into PLC/PRF/5 and HepG2. (**A**) qRT–PCR showed significant overexpression of miR-21 in the transfected cells compared with control cells (^*^P<0.05). (**B**) MTT assay showed that the anti-tumour effects of the combination of IFN-*α* and 5-FU in the miR-21 upregulated cells was significantly lower than in control cells (^*^P<0.05). (**C** and **D**) MTT assay revealed that the anti-tumour effects of IFN-*α* (**C**) and 5-FU (**D**) in the miR-21 upregulated cells was significantly less profound than in control cells (^*^P<0.05). (**E**) The percentage of early apoptotic cells induced by 1000 IU per ml IFN-*α* or 1.0 *μ*g per ml 5-FU among miR-21 upregulated cells was significantly lower than in control cells (^*^P<0.05). Data are mean±s.d. of three experiments.

To evaluate the effect of miR-21 on the response to IFN-*α* and 5-FU, we transfected pre-miR-21 into PLC/PRF/5 and HepG2, which showed the highest and lowest expression level of miR-21 among the five cell lines, respectively. The expression of miR-21 was confirmed to be significantly increased in the transfected cells by qRT–PCR (Figure 2A). The MTT assay showed that cells overexpressing miR-21 were significantly more resistant to the combination therapy of IFN-*α* and 5-FU than the control cells (Figure 2B). Next, we investigated the effect of transfection of pre-miR-21 on the separate growth-inhibitory effect of each of IFN-*α* and 5-FU. The result showed that transfection of pre-miR-21 significantly weakened the growth-inhibitory effect of both IFN-*α* and 5-FU in the two cancer cell lines compared with the control cells (Figure 2C and D). We also evaluated the extent of apoptosis of these cells at 24 h induced by treatment with 1000 IU per ml IFN-*α* or 1.0 *μ*g per ml 5-FU by the Annexin V assay. The percentage of early apoptotic cells was significantly lower in the two cancer cell lines transfected with pre-miR-21 than in control cells (Figure 2E).

**Figure 3:**
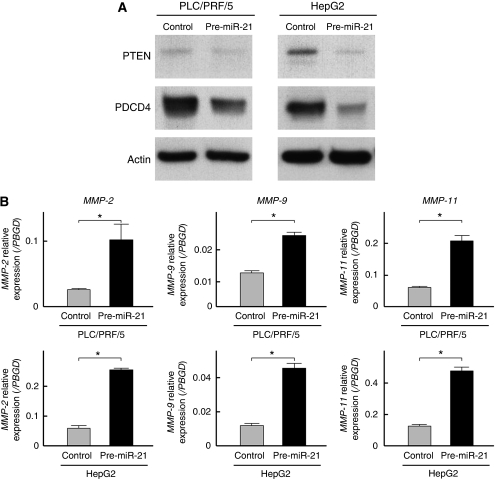
Evaluation of target molecules of miR-21 in PLC/PRF/5 and HepG2 cells transfected with pre-miR-21. (**A**) Western blot analysis demonstrated significant suppression of PTEN and PDCD4 proteins in the transfected cells. (**B**) qRT–PCR showed significant upregulation of *MMP-2*, *MMP-9*, and *MMP-11* mRNAs in the transfected cells (^*^P<0.05). Data are mean±s.d. of three experiments.

Next, the expression levels of PTEN and PDCD4, representing the target molecules of miR-21, were examined by western blot analysis. The expression of these molecules was significantly suppressed in the pre-miR-21-transfected cells (Figure 3A). In addition, the expression levels of *MMP-2*, *MMP-9*, and *MMP-11*, which are also mediated by miR-21, were assessed by qRT–PCR. The results indicated that miR-21 positively modulated the mRNA expression of these *MMPs* (Figure 3B).

### Transfection of anti-miR-21 induces sensitivity to IFN-*α* and 5-FU

**Figure 4:**
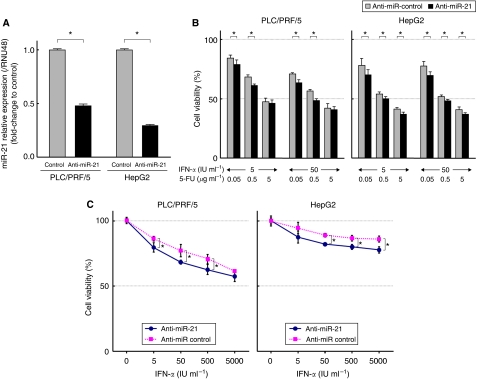

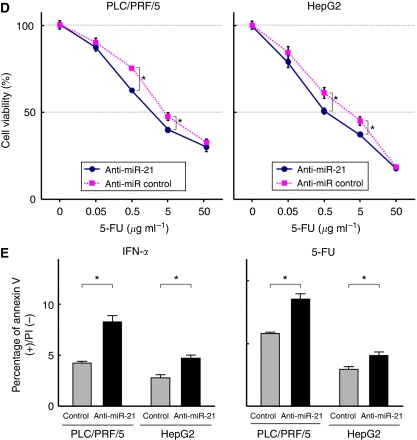
Transfection of anti-miR-21 into PLC/PRF/5 and HepG2. (**A**) The suppression of miR-21 in the transfected cells was confirmed by qRT–PCR (^*^P<0.05). (**B**) MTT assay showed that the anti-tumour effects of the combination of IFN-*α* and 5-FU in the miR-21 upregulated cells was significantly more profound than in control cells (^*^P<0.05). (**C** and **D**) MTT assay showed significantly more anti-tumour effects of IFN-*α* (**C**) and 5-FU (**D**) on the viability of the miR-21 downregulated cells than in control cells (^*^P<0.05). (**E**) Annexin V assay showed that the percentage of early apoptotic cells induced by 1000 IU per ml IFN-*α* or 1.0 *μ*g per ml 5-FU was significantly higher in the miR-21 downregulated cells than in control cells (^*^P<0.05). Data are mean±s.d. of three experiments.

To further assess the effect of miR-21, we transfected anti-miR-21 into PLC/PRF/5 and HepG2. Transfection of cells with anti-miR-21 suppressed miR-21 level compared with the control cells (Figure 4A). The MTT assay showed that the miR-21-suppressed cells were significantly more sensitive to the combination therapy of IFN-*α* and 5-FU than control cells (Figure 4B). Furthermore, the growth-inhibitory effect of a single agent (IFN-*α* or 5-FU) was significantly enhanced in the two cancer cell lines transfected with anti-miR-21 compared with the control cells (Figure 4C and D). In other experiments, Annexin V assay showed significant increase in the percentages of apoptosis of anti-miR-21-transfected cells treated with 1000 IU per ml IFN-*α* or 1.0 *μ*g per ml 5-FU than control cells (Figure 4E).

### PTEN and PDCD4 are responsible for the miR-21-induced resistance

**Figure 5:**
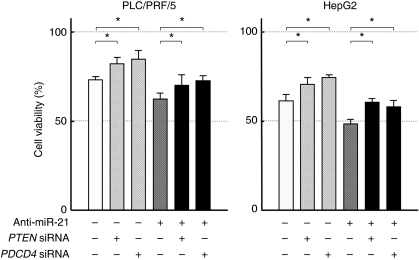
Changes in anti-tumour effects of the combination of IFN-*α* and 5-FU after transfection of anti-miR-21 and/or siRNA against *PTEN* or *PDCD4* in PLC/PRF/5 and HepG2. The MTT assay indicated a weaker anti-tumour effect of 10 IU per ml IFN-*α* and 0.5 *μ*g per ml 5-FU following transfection of *PTEN* or *PDCD4* siRNA, and that the enhanced growth-inhibitory effect by anti-miR-21 transfection was also weakened after the addition of *PTEN* or *PDCD4* siRNA (^*^P<0.05).

We next sought to identify the target molecule responsible for the miR-21-induced resistance. As a potential target molecule, we focused on PTEN and PDCD4, which were confirmed as target molecules by the aforementioned results and also reported previously to be related to apoptosis and drug sensitivity (Jansen *et al*, 2004; Yu *et al*, 2008; Vaidya *et al*, 2009; Li *et al*, 2010). Downregulation of PTEN and PDCD4 expression by their respective siRNAs, PLC/PRF/5, and HepG2 cells became more resistant to the combination therapy (10 IU per ml IFN-*α* and 0.5 *μ*g per ml 5-FU) (Figure 5). In addition, the enhanced growth-inhibitory effect by the aforementioned anti-miR-21 transfection was weakened after the addition of *PTEN* or *PDCD4* siRNA (Figure 5). These findings suggest that PTEN and PDCD4 are responsible, at least in part, for the miR-21-induced resistance.

### MiR-21 expression is associated with clinical response to the IFN-*α*/5-FU combination therapy and prognosis

**Figure 6:**
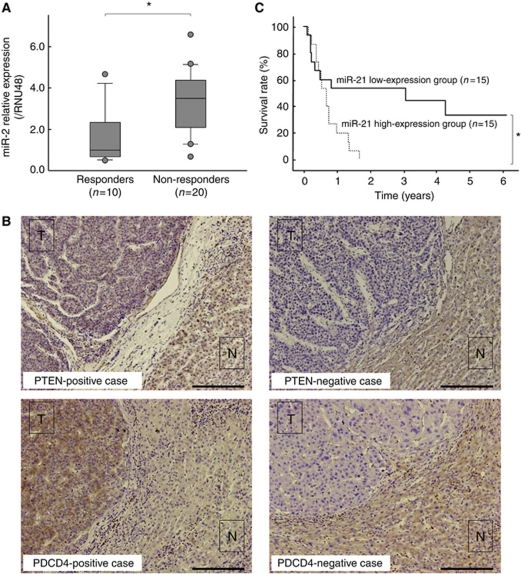
Expression of the miR-21 and its target molecules, PTEN and PDCD4, in tumoural tissue, and the clinical response to the IFN-*α*/5-FU combination therapy in clinical HCC samples. (**A**) The expression of miR-21 in non-responders was significantly higher than in responders (^*^P<0.05). Data are mean±s.d. (**B**) Representative cases of PTEN-positive (*upper left*) or negative (*upper right*) and PDCD4-positive (*lower left*) or negative (*lower right*) (Bar=200 *μ*m) tumours. The expression was identified in the cytoplasm of tumour cells in the positive cases. (**C**) Postoperative overall survival was significantly better in the miR-21 low-expression group than in the miR-21 high-expression group (^*^P<0.05). T=tumour lesion (arrowheads); N=non-tumour lesion.

Next, we examined the relation between miR-21 expression in tumoural tissue and clinical response to the IFN-*α*/5-FU combination therapy. The expression levels of miR-21 in the tumoural tissue varied widely among the patients (Figure 1). A total of 15 patients with values more than the median miR-21 expression level were assigned to the miR-21 high-expression group and the remaining 15 patients were assigned to the miR-21 low-expression group. The clinicopathological factors related to the miR-21 expression status are summarised in Table 1. The data indicate that miR-21 expression did not correlate with any of the clinicopathological factors. We also evaluated the correlation between miR-21 expression level and clinical response to the IFN-*α*/5-FU combination therapy. As shown in Table 2, 13.3% (2/15) of patients of the miR-21 high-expression group were evaluated as responders to the IFN-*α*/5-FU therapy, compared with 53.3% (8/15) of the miR-21 low-expression group, suggesting that the miR-21 expression was significantly associated with the clinical response to the IFN-*α*/5-FU combination therapy (*P*=0.0201). In other words, miR-21 expression was significantly higher in non-responders than in responders (*P*=0.0109, Figure 6A). The sensitivity, specificity, and accuracy for the prediction of the response to IFN-*α*/5-FU therapy by miR-21 expression were 80.0% (8/10), 65.0% (13/20), and 70.0% (21/30), respectively.

Next, we examined PTEN and PDCD4 expression by immunohistochemistry using clinical specimens from the 30 patients. Staining for PTEN and PDCD4 was noted in the cytoplasm of tumour cells of samples of 8 and 11 patients, respectively (Figure 6B). Although there was no significant association between PTEN expression and miR-21 expression, the expression of PDCD4 tended to correlate with that of miR-21 (Table 3). Neither PTEN nor PDCD4 expression was significantly associated with the response to the IFN-*α*/5-FU combination therapy (Table 3). These results suggest that analysis of miR-21 expression is more useful for predicting the response to the combination therapy than that of the two representative target molecules, PTEN and PDCD4.

Finally, we examined the relationship between miR-21 expression and prognosis. The overall survival rate of the miR-21 low-expression group was significantly better than that of the miR-21 high-expression group (*P*=0.0250, Figure 6C). These results suggest that miR-21 expression in HCC tissues is a useful marker for prediction of the clinical response to the combination therapy and prognosis.

## Discussion

In this study, we analysed the expression of miR-21 in HCC cell lines and clinical HCC samples. Previously, Meng *et al* (Meng *et al*, 2007) reported a significantly high expression of miR-21 in HCC cells and that miR-21 contributed to cell proliferation, migration, and invasion. Although we did not examine migration or invasion of HCC cells transfected with miR-21, we confirmed significant increase in proliferation of cells transfected with miR-21 compared with control cells (data not shown), in agreement with the previous report of Meng *et al* (2007). To our knowledge, however, there are no reports on the correlation between miR-21 expression and chemoresistance in HCC. In this study, we found a significant relationship between miR-21 expression and chemoresistance in HCC.

Several investigators have reported the correlation of miR-21 expression with chemoresistance in pancreatic cancer, cholangiocarcinoma, and glioblastoma (Meng *et al*, 2006; Li *et al*, 2009; Moriyama *et al*, 2009; Park *et al*, 2009). The result of this study that miR-21 expression was associated with chemoresistance in HCC was consistent with these previous reports. However, few of the above reports examined the underlying mechanism of the miR-21-induced chemoresistance. In the majority of the above reports on miR-21-induced chemoresistance, miR-21 induced changes in the expression of target molecules deemed potentially responsible for the chemoresistance. However, these studies did not evaluate the change in chemoresponsiveness after manipulation of the expression of the target molecules. For example, Meng *et al* (2007) reported that miR-21 inhibited gemcitabine-induced apoptosis by negatively regulating PTEN and its downstream pathway, based on previous reports of the association between PTEN expression and chemosensitivity (Yu *et al*, 2008; Vaidya *et al*, 2009). Other studies reported miR-21-induced chemoresistance by downregulation of PDCD4 proteins, on the basis of previous reports of the relation between PDCD4 and chemosensitivity (Jansen *et al*, 2004; Bourguignon *et al*, 2009). Moriyama *et al* (2009) also reported miR-21 induced chemoresistance to gemcitabine and changes in MMPs expression, and speculated that these miR-21-induced changes in chemoresistance were mediated through MMPs, based on previous reports that the miR-21 indirectly induced MMPs expression (by negative regulation of tissue inhibitor of metalloproteinases 3 (TIMP3) and reversion-inducing cysteine-rich protein with Kazal motifs (RECK)) and that MMPs levels correlated significantly with chemosensitivity (Gabriely *et al*, 2008; Almendro *et al*, 2009; Song *et al*, 2009). On the other hand, in addition to the confirmation of miR-21-induced chemoresistance and changes in the aforementioned target molecules in pre-miR-21-transfected cells including PTEN, PDCD4, and MMPs, we also demonstrated that the miR-21-induced changes in chemoresponse were ameliorated by downregulation of PTEN or PDCD4 by the respective siRNA. Thus, our results suggest that miR-21 induces chemoresistance to IFN-*α* and 5-FU, mediated through PETN and PDCD4. Furthermore, we also confirmed the association between miR-21 expression and response to the combination therapy in clinical HCC samples. Our analysis demonstrated that miR-21 expression, but not PTEN or PDCD4, correlated significantly with the response to the combination therapy. It was noteworthy that the expression levels of PTEN and PDCD4 tended to correlate inversely with that of miR-21 in tumour tissues. This discrepancy suggests that the expression of both PTEN and PDCD4 is under the control of not only miR-21 but also their mRNAs and/or those of various posttranslational modulators including other miRs. In general, miRs modulate the expression of multiple target molecules, suggesting there are possibly other unknown target molecules of miR-21 responsible for the chemoresistance other than PTEN and PDCD4. Taken together, determination of miR-21 expression rather than various target molecules provides a better prediction of the response to the combination therapy.

We reported previously that IFNAR2 and epithelial cell adhesion molecule (EpCAM) correlate significantly with the clinical response to the IFN-*α*/5-FU combination therapy (Ota *et al*, 2005; Nagano *et al*, 2007a; Noda *et al*, 2009). Therefore, in this study, we investigated the effects of pre-miR-21 transfection on the expression status of IFNAR2 and EpCAM. The result showed no significant change in the expression status (data not shown), suggesting that the chemoresistance induced by miR-21 is different from the relationship between the anti-tumour effect and IFNAR2 and EpCAM expression.

In summary, the results of this study demonstrated a significant association between the miR-21 expression and the response to IFN-*α* and 5-FU in HCC cell lines in genetic manipulation experiments. Moreover, this significant correlation was also confirmed in human clinical HCC samples. Our findings suggest that the miR-21 could be a potentially useful marker for the prediction of the clinical response to the IFN-*α*/5-FU combination therapy, and that the miR-21 may serve as a potential target for HCC therapy.

## Figures and Tables

**Table 1:** Correlation between clinicopathological factors and miR-21 expression status

	**miR-21 expression**		
	**High (*n*=15)**	**Low (*n*=15)**	***P*-value**
Age (years)^a^	54.2±9.3	58.1±13.4	0.3669
Gender (male/female)	13/2	14/1	>0.9999
Child-Pugh classification (A/B)	9/6	10/5	0.7048
AFP (ng ml^−1^) (<400/⩾400)	5/10	6/9	0.7048
PIVKA-II (mAU l^−1^) (<1000/⩾1000)	1/14	5/10	0.1686
Histological grade (mod/poor/undifferentiated)	0/14/1	1/12/2	0.4754
IFNAR2 status (±)	5/10	5/10	>0.9999

Abbreviations: AFP=*α*-fetoprotein; IFNAR2=type I interferon receptor type 2; miR=microRNA; mod=moderately differentiated; PIVKA-II=protein induced by vitamin K absence or antagonists-II; poor=poorly differentiated.

a Data are mean±s.d.

**Table 2:** Association between miR-21 expression and clinical response to the combination therapy

	**Responders**	**Non-responders**	***P*-value**
miR-21 high expression (*n*=15)	2	13	0.0201
miR-21 low expression (*n*=15)	8	7	

Abbreviation: miR=microRNA.

**Table 3:** Association of PTEN and PDCD4 expression with miR-21 expression and clinical response to the combination therapy

	**miR-21 expression**			**Clinical response**		
	**High**	**Low**	***P*-value**	**Responders**	**Non-responders**	***P*-value**
*PTEN*						
(+)	2	6	0.2148	4	4	0.3841
(−)	13	9		6	16	
						
*PDCD4*						
(+)	3	8	0.0582	4	7	>0.9999
(−)	12	7		6	13	

Abbreviations: miR=microRNA; PDCD4=programmed cell death 4; PTEN=phosphatase and tensin homolog.
